# Designing Effective Visual Feedback for Facial Rehabilitation Exercises: Investigating the Role of Shape, Transparency, and Age on User Experience

**DOI:** 10.3390/healthcare11131835

**Published:** 2023-06-23

**Authors:** Sojung Gwak, Kyudong Park

**Affiliations:** 1 Department of Artificial Intelligence Applications, Kwangwoon University, Seoul 01897, Republic of Korea; 2 School of Information Convergence, Kwangwoon University, Seoul 01897, Republic of Korea

**Keywords:** facial expression recognition, visual feedback design, mobile application, interaction design, human–AI interaction

## Abstract

Facial expression recognition technology has been utilized both for entertainment purposes and as a valuable aid in rehabilitation and facial exercise assistance. This technology leverages artificial intelligence models to predict facial landmark points and provide visual feedback, thereby facilitating users’ facial movements. However, feedback designs that disregard user preferences may cause discomfort and diminish the benefits of exercise. This study aimed to develop a feedback design guide for facial rehabilitation exercises by investigating user responses to various feedback design methods. We created a facial recognition mobile application and designed six feedback variations based on shape and transparency. To evaluate user experience, we conducted a usability test involving 48 participants (24 subjects in their 20s and 24 over 60 years of age), assessing factors such as feedback, assistance, disturbance, aesthetics, cognitive ease, and appropriateness. The experimental results revealed significant differences in transparency, age, and the interaction between transparency and age. Consequently, it is essential to consider both transparency and user age when designing facial recognition feedback. The findings of this study could potentially inform the design of more effective and personalized visual feedback for facial motion, ultimately benefiting users in rehabilitation and exercise contexts.

## 1. Introduction

Computer vision, as defined by Learned-Miller [1], is a scientific discipline that aims to grant visual perception capabilities to computers and other machines. By recognizing human faces through cameras, artificial intelligence models utilizing computer vision techniques can predict facial landmark points, which in turn can serve as a foundation for implementing and displaying visual feedback to users. 

The computer vision field has significantly advanced in recent years, leading to the development of innovative facial expression recognition technologies. Technologies that utilize cameras and visual feedback designs have been employed for various purposes, including entertainment applications such as camera filters and augmented reality (AR) emojis, and facial expression enhancement [2,3]. Furthermore, these technological advancements have proven useful in the rehabilitation and treatment of patients with facial dysfunctions [4].

Facial dysfunction, a condition that affects individuals with Bell’s palsy, Parkinson’s disease, and other related disorders, often results in difficulty expressing emotions and fulfilling social functions that require these expressions [5,6]. In this context, facial exercises have been demonstrated to be effective in the treatment of facial paralysis [7]. Various methods for facilitating recovery by incorporating facial movements have been explored in the facial rehabilitation field [8].

Visual feedback is a valuable tool for assisting users in performing facial exercises. However, the efficacy of these exercises is contingent on the design of the visual feedback provided [9]. Inadequate feedback designs that do not consider user preferences may lead to discomfort and diminish the benefits of the exercise. Therefore, establishing a comprehensive understanding of user responses to different feedback design approaches is crucial.

In this study, we aim to develop a feedback design guide tailored for facial rehabilitation exercises by examining user responses based on different feedback design methods. To achieve this goal, we formulated the following research questions:

- RQ1: How do variations in the feedback design shape influence user responses?
- RQ2: Are there any differences in user response based on the transparency of the feedback design?
- RQ3: Do user responses to feedback design differ across age groups?

By addressing these research questions, we hope to provide valuable insights into the development of effective feedback design strategies for facial rehabilitation exercises. In turn, this could enhance the overall experience and outcomes for patients undergoing facial rehabilitation treatments. By leveraging the capabilities of computer vision technology and considering user preferences, we believe that it is possible to develop more effective and personalized rehabilitation strategies for individuals with facial dysfunctions. With advancements in the field of facial expression recognition, researchers are exploring the potential of incorporating machine learning algorithms and deep learning techniques to enhance the accuracy of facial landmark detection and overall user experience [10].

The integration of computer vision and facial expression recognition technology has shown potential in various applications beyond rehabilitation, such as emotion recognition and human–computer interaction [11,12]. Understanding user preferences and responses to different feedback design methods has broad implications for the development of user-friendly and effective applications in these fields [13].

In summary, this study contributes to ongoing efforts to improve facial rehabilitation treatment by examining user responses to various feedback design methods. Our findings can inform the development of more effective personalized feedback designs that cater to the needs and preferences of individuals with facial dysfunctions.

## 2. Related Work

### 2.1. Face Landmark Detection

Many facial landmark detection techniques have been developed to date. Wu and Ji [14] classified facial landmark algorithms into three categories: holistic methods, Constrained Local Model (CLM) methods, and regression-based methods. Holistic approaches involve constructing a comprehensive model that represents the complete shape and patterns of the face. The Active Appearance Model (AAM) is a basic model using holistic methods. Cootes et al. [15] suggested an AAM constructed based on an understanding of the correlation between variations in the model parameters and the resulting image inaccuracies.

Constrained Local Models (CLMs) utilize a global shape model and construct a local shape model. The process of constructing the CLM model bears a resemblance to the AAM approach. However, rather than modeling the entire object region, CLM focuses on creating a collection of local feature templates [16]. 

Regression-based techniques implicitly capture both facial shape and appearance information. In regression-based methods, cascaded regression minimizes the impact of outliers by explicitly detecting occlusions and employing resilient shape-indexed features [17]. With the deep structures of convolutional networks, it is possible to extract features from the entire face area well in the initial stage [18]. 

More recently, various models, such as STAR, AnchorFace, HRPNet, and 3DDE, have been used to execute facial landmark detection [19,20,21,22].

### 2.2. Rehabilitation with Visual Feedback

Several studies have shown that visual feedback can help patients and users during rehabilitation and exercise. A study was conducted to propose an easy-to-understand color-based visual feedback for stroke patients, showing promising results [23]. Another study showed that motor rehabilitation based on augmented visual feedback is useful for people with Parkinson’s disease [24].

In addition, there have been studies related to facial rehabilitation exercises. Delannoy and Ward [25] proposed a vision-based facial exercise system for people who need facial exercises. It tracks key facial features to provide basic visual feedback, with the expectation that it could be more conducive to rehabilitation at home. In another study, Barrios Dell’Olio and Sra proposed FaraPy, the first mobile augmented reality mirror therapy system for facial paralysis that provides real-time visual feedback and tracks the user’s paralysis progress over time. It was highly favored by users [26].

## 3. Materials and Methods

To accomplish our study’s aims, we conducted an experiment and collected questionnaires and feedback from participants. This was a mixed-methods study, as it included both qualitative and quantitative data.

### 3.1. Participants

Forty-eight participants, including 24 individuals in their 20s (16 males and 8 females aged 23.6 ± 1.3) and 24 individuals aged 60 and over (3 males and 21 females, aged 74.2 ± 6.9) participated in the experiment voluntarily. Subjects in their 20s were recruited from Kwangwoon University, Seoul, South Korea and subjects aged 60 years and over were recruited with the help of a welfare center in Seoul, South Korea. All participants had been using smartphones for more than a year. To minimize the learning and order effects, we counterbalanced the six treatment conditions (feedback designs) using a Balanced Latin Square. The study was conducted in adherence with the procedures approved by the Institutional Review Board (IRB) of Kwangwoon University, as evidenced by approval number: 7001546-202300425-HR(SB)-004-01. Participants were furnished with a detailed guide encompassing ethical considerations alongside an extensive research overview document. The researcher conscientiously apprised the participants about the possible benefits as well as potential side effects associated with the experiment. It was explicitly communicated that participation was voluntary and the participants could opt for discontinuation or a break at their discretion. Before their involvement in the study, all participants expressed their consent by signing an informed consent document. Upon completion of the study, a remuneration of KRW 30,000 was disbursed to each participant as a token of appreciation for their time and participation.

### 3.2. Experimental Design

**Figure 1:**
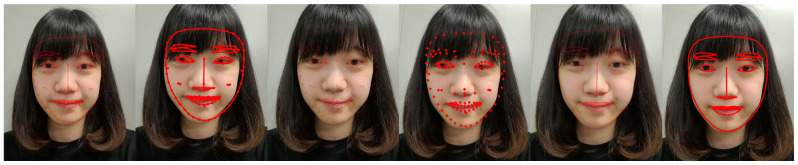
Six treatment conditions (points with lines + translucent, points with lines + opaque, points + translucent, points + opaque, lines + translucent, lines + opaque).

We developed a facial recognition mobile application that incorporated six feedback designs based on shape (points, lines, and points with lines) and transparency (opaque versus translucent) (Figure 1).

Through a literature review, Yu and Kim [27] identified five critical evaluation factors for mobile healthcare application graphical user interface (GUI) design usability: efficiency, cognitive ease, aesthetics, feedback, and consistency. In this study, we selected three of these factors, feedback, aesthetics, and cognition, which we deemed relevant for evaluating feedback design. We added three additional factors—help, disturbance, and appropriateness—to create a set of six dependent variables (Table 1). Because appropriateness is included in the five criteria for good design, we used appropriateness of elements as an evaluation metric [28], and we included help and disturbance as dependent variables to directly ask whether the feedback design was helpful or disturbing to rehabilitation.

The feedback questions were formulated based on Jakob Nielsen’s user interface (UI) design principle, which emphasizes the provision of prompt responses and visual or audible signals during task execution. The aesthetics question assessed whether the interface design elements created a visually harmonious and concise impression in line with Nielsen’s “consistency and standards” usability evaluation principle [29]. The cognitive ease question was derived from Nielsen’s “recognition rather than recall” principle, which aims to minimize cognitive effort for users.

### 3.3. Apparatus

We developed the core functions of the application using Google’s ML Kit Face Detection API [30]. The application programming interface (API) detects faces within an input frame and outputs up to 133 Landmark Points in 2D coordinates. 

In this study, we implemented the feedback design as a treatment condition by displaying it on a user’s face on a screen based on the corresponding coordinate values. The API provides a model for predicting the probability of a smiling face with closed eyes in a frame. We used this model to implement a function that detects “laughing” and “winking” when the user looks at the camera.

### 3.4. Task and Procedure

The experiment was conducted in a quiet and unobtrusive laboratory setting. Participants were asked to perform tasks that involved holding “laughing” and “winking” expressions for 3 s each and repeating the process four times for each of the six treatment conditions.

First, participants provided informed consent by signing consent forms after being briefed on the study’s objectives. Thereafter, they were guided through the experimental procedure, provided with an overview of the application, and instructed on the research methods. Subsequently, they proceeded with the experiment according to their assigned task orders. After completing each of the six tasks, the participants filled out a questionnaire on feedback, aesthetics, cognitive ease, help, disturbances, and appropriateness. In addition to measuring specific evaluation attributes, we conducted follow-up interviews to better understand which designs were most helpful in expressing emotions and which were perceived as uncomfortable or disruptive. The entire process took approximately 60 min per participant.

### 3.5. Data Collection and Analysis

Data were collected in a self-reported format with scores ranging from 0 to 100 for each of the questions in Table 1. We conducted repeated-measures ANOVA on a mixed-subjects design with more than two factors, measuring feedback, aesthetics, cognitive ease, help, disturbance, and appropriateness as dependent variables. Normality and sphericity assumptions were tested, and the results indicated that these assumptions were met. We used Jamovi 2.3.21 for statistical analysis.

## 4. Results

The study participants’ demographic information is shown in Table 2.

### 4.1. Quantitative Analysis

#### 4.1.1. Feedback

For the feedback question (“Can you clearly check the face condition and results?”) the grand mean score was 72.05. The score for those in their 20s was 64.67, while for those over 60 years old it was 79.43. For the main effect, transparency, the score for translucency was 77.02 and that for opacity was 67.08. We found significant differences for transparency (F_1,46_ = 7.9667, *p* < 0.01, partial η² = 0.148), age (F_1,46_ = 10.4, *p* < 0.01, η² = 0.086), and the interaction between transparency and age (transparency × age) (F_1,46_ = 10.1, *p* < 0.01, partial η² = 0.18), with *p*-values less than 0.01 (Table 3).

#### 4.1.2. Aesthetics

Regarding the aesthetics question (“Do you think the visual elements are composed in harmony?”) the grand mean score was 71.06. The score for those in their 20s was 60.87, whereas for those over 60 years old it was 81.25. The translucency score was 74.75 and the opacity score was 67.37. We observed significant differences for transparency (F_1,46_ = 8.199, *p* < 0.01, partial η² = 0.151), age (F_1,46_ = 17.1, *p* < 0.001, η² = 0.167), and the interaction between transparency and age (transparency × age) (F_1,46_ = 13.088, *p* < 0.001, partial η² = 0.221) (Table 3).

#### 4.1.3. Cognitive Ease

For the cognitive ease question (“Was it possible to make a facial expression with less cognitive effort?”) the grand mean score was 72.48. The score for those in their 20s was 62.92, while for those over 60 years old it was 82.05. For the main effect, transparency, the translucency score was 76.6 and the opacity score was 68.37. We identified significant differences for transparency (F_1,46_ = 9.4589, *p* < 0.01, partial η² = 0.171), age (F_1,46_ =14.0, *p* < 0.001, η² = 0.148), and the interaction between transparency and age (transparency × age) (F_1,46_ = 10.60444, *p* < 0.01, partial η² = 0.187) (Table 3).

#### 4.1.4. Help

For the help question (“Do you think you were helped by visual elements when making a presented expression?”) the grand mean score was 64.75. The score for those in their 20s was 53.38, while for those over 60 years old it was 76.12. For transparency, the translucency score was 67.75 and the opacity score was 61.75. We detected significant differences with *p*-values less than 0.05 for age (F_1,46_ = 21.5, *p* < 0.001, η² = 0.18), transparency × age (F_1,46_ = 8.224, *p* < 0.01, partial η² = 0.152), and shape × age (F_2,92_ = 3.285, *p* < 0.05, partial η² = 0.067) (Table 3).

#### 4.1.5. Disturbance

Regarding the disturbance question (“Do you think visual elements were disturbed when making facial expressions?”) the grand mean score was 58.95. The score for those in their 20s was 60.82, while for those over 60 years old it was 57.08. For transparency, the translucency score was 66.2 and the opacity score was 51.7. We found significant differences with *p*-values less than 0.05 for transparency (F_1,46_ = 18.840, *p* < 0.001, partial η² = 0.291), transparency × age (F_1,46_ = 12.330, *p* < 0.05, partial η² = 0.211), and transparency × shape × age (F_2,92_ = 7.884, *p* < 0.001, partial η² = 0.146) (Table 3).

#### 4.1.6. Appropriateness

For the appropriateness question (“Do you think it is appropriately composed of visual elements that are essential when making expressions?”) the grand mean score was 71.99. The score for those in their 20s was 65.62, while for those over 60 years old it was 78.37. The translucency score was 74.25 and the opacity score was 69.73. We discovered significant differences with *p*-values less than 0.05 for transparency (F_1,46_ = 4.939, *p* < 0.05, partial η² = 0.097), age (F_1,46_ = 5.99, *p* < 0.05, η² = 0.07), transparency × age (F_1,46_ = 5.568, *p* < 0.05, partial η² = 0.108), and transparency × shape (F_2,92_ = 6.204, *p* < 0.01, partial η² = 0.119) (Table 3).

## 5. Discussion

### 5.1. Effect of Shapes in the Feedback Design

Regarding the shape of the feedback design, no significant differences were observed for any of the six dependent variables in the quantitative analysis. This suggests that shape preferences were likely driven by personal taste rather than visual assistance. Consequently, we found no difference in the user responses based on the shape of the feedback design, answering RQ1 (Is there a difference in the user’s response according to the shape of the feedback design?).

### 5.2. Effect of Transparency in the Feedback Design

Regarding transparency, significant differences were found for the five dependent variables, with higher scores for translucent designs than for opaque designs. This confirms differences in user responses to RQ2 (Is there a difference in the user response regarding the transparency of the feedback design?). Overall, considering the higher scores for translucent designs, it is advisable to opt for a translucent design.

### 5.3. Effect of Age

**Figure 2:**
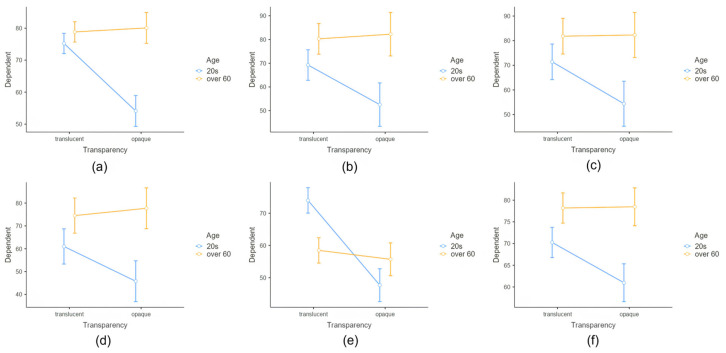
Interaction effects of (**a**) Feedback, (**b**) Aesthetics, (**c**) Cognitive Ease, (**d**) Help, (**e**) Disturbance, and (**f**) Appropriateness between Factor Transparency and Factor Age on Dependent Variable score. Error bars represent standard errors of the mean.

The interaction between age and transparency was significant for all six dependent variables (Figure 2). Post hoc analysis revealed significant differences between translucent/20s and opaque/20s designs for all six dependent variables (*p* < 0.05), and between opaque/20s and opaque/60s for five dependent variables (*p* < 0.05). Translucent designs scored higher than opaque designs for participants in their 20s, while opaque designs scored higher for participants over 60 years old. The qualitative interview results were aligned with those of the quantitative analysis. Participants in their 20s found opaque designs uncomfortable because they obscured their facial expressions, whereas participants aged over 60 years found opaque designs helpful in delineating facial contours.

Age and shape interactions were significant for only one dependent variable. However, interviews revealed age-dependent shape preferences. Participants in their 20s favored designs with points and lines, citing improved facial expression recognition. In contrast, the preferences among participants over 60 years old were more diverse.

These findings indicate a difference in user responses to RQ3 (Is there a difference in the user’s response to the feedback design according to age?). For participants over 60 years old, the scores for opaque feedback designs were higher than those for participants in their 20s; therefore, it is recommended that user age should be considered when selecting transparency.

This study aimed to develop a feedback design guide for facial rehabilitation exercises by examining user responses to different feedback design methods. We developed facial recognition mobile applications and designed six feedback designs based on shape (points, lines, and points with lines) and transparency (opaque vs. translucent). We then assessed user experience in terms of feedback, aesthetics, cognitive ease, help, disturbance, and appropriateness across 48 participants in two age groups (users in their 20s and users over 60).

### 5.4. Implications

Older adults’ negative attitudes toward technology are most often associated with discomfort, security, and reliability issues [31]. This is consistent with our findings that for older adults the combination of visual feedback on the screen should be simple and comfortable to use.

Recently, an increasing viewpoint arguing that what users perceive as good design is highly dependent on personal preference calls into question the feasibility of universal design guidelines [32]. Therefore, in this case it is a good idea to let users decide their own feedback design preferences. Our research shows that transparency should be a factor in user-defined visual feedback designs, and that the age of the user has a major impact on their preferences.

## 6. Conclusions

We found no significant difference in user responses regarding the different feedback design shapes. Thus, shape preference appears to be driven by personal taste rather than by visual assistance. However, we observed significant differences in user responses concerning transparency. Translucent designs scored higher overall, suggesting that they were generally preferred. Notably, age played a crucial role in modulating user preferences for feedback design transparency. Younger participants favored translucent designs, whereas older participants preferred opaque designs.

Based on these results, we recommend that future feedback designs for facial rehabilitation exercises should generally lean toward translucent designs. However, designers should consider user age when determining the level of transparency, as older users may benefit more from opaque designs. This study’s findings contribute to a better understanding of user preferences in feedback design, and can help to inform the development of more effective and user-friendly facial rehabilitation applications.

Future studies could investigate additional factors that influence user preferences, such as cultural background, gender, and previous experience with similar technologies. People who have a cultural aversion to making facial expressions, or who are not good at expressing emotions through facial expressions, may provide lower usability evaluation scores due to their antipathy to the task itself, making the scores less reliable. Therefore, usability evaluation of visual feedback designs should be conducted with action-unit tasks such as opening the mouth or raising the eyebrows, rather than winking, smiling, etc. Additionally, examining the long-term effects of different feedback designs on user engagement and adherence to facial rehabilitation exercises could provide valuable insights for practical applications.

## Figures and Tables

**Table 1:** Usability Assessment Criteria Items.

Dependent Variable	Detailed Evaluation Attributes [0–100 Points]
Feedback	When you make an expression, do the visual elements (points/lines) react immediately?
Aesthetic	Do you think the visual elements are organized harmoniously?
Cognitive Ease	Were you able to imitate facial expressions with little cognitive effort?
Help	Do you think you were helped by visual elements when making the suggested facial expressions?
Disturbance	Do you think the visual elements were interrupted when making expressions?
Appropriateness	Do you think that it is properly composed of only the visual elements that are necessary to imitate facial expressions?

**Table 2:** Participants’ demographic information.

		N	Percent (%)
Gender	Male	19	39.6
	Female	29	60.4
Age	20 s	24	50
	Over 60	24	50
Ethnicity	Korean	48	100

**Table 3:** *p*-value for Shape, Transparency, and Age by Dependent Variable.

Effect	Feedback	Aesthetics	Cognitive Ease	Help	Disturbance	Appropriateness
Shapes	0.087	0.193	0.774	0.46	0.277	0.216
Transparency	<0.01 **	<0.01 **	<0.01 **	0.067	<0.001 ***	<0.05 *
Age	<0.01 **	<0.001 ***	<0.001 ***	<0.001 ***	0.5	<0.05 *
Shapes × Age	0.958	0.11	0.998	<0.05 *	0.493	0.174
Transparency × Age	<0.01 **	<0.001 ***	<0.01 **	<0.01 **	<0.01 **	<0.05 *
Shapes × Transparency	0.569	0.12	0.995	0.099	0.093	<0.01 **
Shapes × Transparency × Age	0.097	0.656	0.293	0.698	<0.001 ***	0.632

*, **, *** means *p*-value is lower than 0.05, 0.01 and 0.001 respectively.

## Data Availability

The data presented in this study are available on request from the corresponding author. While we made every effort to obtain consent for data sharing during the informed consent process, some participants expressed concern about the potential risks of sharing their information publicly. We respect their decision, and as a result we cannot provide public access to these data.
